# Physical Activity and Physical Competence in Overweight and Obese Children: An Intervention Study

**DOI:** 10.3390/ijerph17176370

**Published:** 2020-09-01

**Authors:** Milena Morano, Claudio Robazza, Laura Bortoli, Irene Rutigliano, Montse C. Ruiz, Angelo Campanozzi

**Affiliations:** 1 Parisi-De Sanctis Institute, MIUR (Italian Ministry of Education, University and Research), 71121 Foggia, Italy; milenamorano@gmail.com; 2 School of Medicine and Health Sciences, “G. d’Annunzio” University of Chieti-Pescara, 66013 Chieti, Italy; 3 BIND-Behavioral Imaging and Neural Dynamics Center, Department of Medicine and Aging Sciences, “G. d’Annunzio” University of Chieti-Pescara, 66013 Chieti, Italy; l.bortoli@unich.it; 4 Pediatrics, IRCCS Casa Sollievo della Sofferenza, 71013 San Giovanni Rotondo, Italy; irene.rutigliano@libero.it; 5 Faculty of Sport and Health Sciences, University of Jyväskylä, 40014 Jyväskylä, Finland; montse.ruiz@jyu.fi; 6 Pediatrics, Department of Medical and Surgical Sciences, University of Foggia, 71122 Foggia, Italy; angelo.campanozzi@unifg.it

**Keywords:** obesity, school-based intervention, actual physical abilities, fitness, perceived physical abilities, healthy habits

## Abstract

With the current obesity epidemic and the decline of fitness among school-aged children, the importance of obesity interventions to promote physical activity and healthy habits has become indisputable. The purpose of this study was to assess the efficacy of a school-based multicomponent intervention in increasing physical activity (PA) levels, actual physical abilities, and perceived physical abilities in clinical and nonclinical samples of overweight/obese boys and girls aged 10–12 years. The clinical intervention group (*n* = 35) participated in a 7-month after-school program in addition to curricular physical education lessons, while the nonclinical control group (*n* = 29) received usual curricular lessons. Measures included levels of PA and fitness and individual’s perceptions of physical ability. After treatment, the intervention group showed improved PA levels, perceived physical ability, and throwing and jumping task performances compared to the control group. Results indicate that a multicomponent program can improve levels of PA, fitness, and perceived competence of overweight participants. Findings highlight the importance of a comprehensive healthy lifestyle program that includes physical, psychosocial, and behavioral factors and suggest practical implications for educators, trainers, and teachers in identifying best practices targeting childhood obesity.

## 1. Introduction

Childhood overweight and obesity are known to affect physical and psychosocial health and quality of life [1,2]. Increasing weight status has an adverse effect on perceived physical competence (i.e., individual’s perception of physical condition and sport competence), mainly due to movement difficulties, including poor performance on body-mass dependent motor tasks [2,3,4,5]. Cross-sectional studies have demonstrated that obese children perceive themselves as less physically competent, perform poorly on weight-bearing tasks [6,7,8] and, consequently, are usually less physically active than their non-obese counterparts [9]. On the other hand, improved actual motor skill competence is related to increased physical fitness [10], higher perceived physical competence [7], and increased motivation toward physical activity (PA; [11]). Recently, researchers have successfully begun to implement interventions that employ an evidence-based model of physical literacy (i.e., a multidimensional framework that refers to movement competence, affect, confidence, and motivation toward PA; [12]) as a health determinant of school-aged children through participation in PA [13,14,15]. Notwithstanding this evidence, little experimental or longitudinal research has explored the benefits of improved actual and perceived physical competence on physical and psychosocial difficulties of obese children [1,2].

Different theories of motivated behavior applied to understanding and predicting PA participation (e.g., competence motivation theory, self-efficacy theory) highlight perceived competence as one of the most consistent predictors of PA [16,17]. Children with low levels of actual and perceived physical competence, such as obese children, may be less motivated to engage and persist in physical activities, resulting in fewer movement opportunities to improve their skillfulness and perception of competence [18]. Conversely, if children develop adequate levels of actual motor competence over time, both actual competence and perception of competence can increase PA and fitness level [19,20]. Perceived skill competence is deemed to mediate the relationship between actual competence and PA levels [21] also in clinically obese children [22]. However, whether and how low perceived physical competence of overweight children may influence their motivation to participate in regular exercise is less clear [8]. Furthermore, little is known about the effects of obesity interventions on the specific determinants of PA and fitness and how such factors may interact to improve physical and psychosocial health among obese children. Findings from previous studies suggest that psychosocial factors are potential targets to enhance physical fitness [23] or to promote PA levels [24] and emphasize the importance of considering such factors within interventions involving obese children [25]. More clearly identified relations between some hypothesized mediators (e.g., self-efficacy, body image, perceived competence, and enjoyment) and PA could be important in developing optimally effective exercise programs [26,27].

A recent systematic review suggests that school-based interventions can greatly contribute to prevent obesity and to promote PA and fitness if the programs are multicomponent; centered on PA; and focused on the content, quality, and duration [28]. Findings of a cross-sectional study on a large sample of students aged 6–18 years showed a physical activity-friendly school environment to be associated with lower risk of obesity [29]. A meta-analysis found that PA interventions underpinned with relevant theoretical views (e.g., social cognitive theory, health promotion model, social learning theory) and that include multiple components are the most promising in the school setting [30]. These approaches should include evaluations of some additional aspects, such as motivation and physical self-competence, as intervention outcomes addressing childhood obesity [1,28]. Unfortunately, despite evidence of the psychological benefits of increased PA [31,32], few obesity interventions have taken into account psychosocial determinants of PA behavior [5,33,34]. The majority of such intervention studies have shown conflicting results regarding the relationship between weight status and psychological improvements, suggesting that psychosocial benefits of increased PA in obese children are not always related to weight reduction [1,31]. Moreover, most published obesity interventions in school settings have been limited to either clinical or nonclinical groups of children but failed to include both groups at the same time.

Therefore, the purpose of this study was to examine, in overweight and obese children, the impact of a multicomponent program, including health education and supervised exercise training, on PA levels, physical fitness, and perceived competence. Perceived competence is a key construct in many theoretical models of achievement motivation and a major focus of concern in different educational settings [35]. Particularly, competence perceptions have been often considered in contexts in which competence is highly visible, such as the physical domain, including physical education (PE) and structured PA and exercise. In particular, the aim of our research was to evaluate the effect of a 7-month school-based intervention targeting PA levels and actual and perceived physical abilities in both clinical and nonclinical samples of overweight boys and girls. We expected an extracurricular activity program, based on enjoyable physical experiences, skill improvement, and physical training, to be more effective in improving fitness, motor competence, PA levels, and perceived competence of a clinical group compared to a standard PE program alone.

## 2. Materials and Methods

### 2.1. Participants and Procedures

Data for this study were derived from a larger project, named “Healthy Lifestyles Intervention for Obese Schoolchildren (HeLIOS)” [5,36]. A total of sixty-four overweight/obese children (36 boys and 28 girls, aged 11.3 ± 0.5 years) participated in the present study. The clinical intervention group consisted of 35 children who attended the outpatient hospital clinic of a pediatric obesity center in Puglia, one of the regions in southern Italy most affected by pediatric obesity [37]. Of the total clinical sample considered, about 85% agreed to participate in a 7-month after-school multicomponent program that included health education, PA, and exercise training. The nonclinical control participants consisted of 29 children recruited in an urban middle school located in the same region. Of the nonclinical sample, about 90% agreed to participate in the study and followed the usual curricular PE lessons without taking part in planned extracurricular activities.

Children aged 10 to 12 years were considered eligible under the following criteria: (a) their body mass index (BMI) was within or above the 85th percentile of the age- and gender-specific values on the Centers for Disease Control and Prevention (CDC) growth charts [38]; (b) they did not have any underlying conditions that would impede their participating in PA; and (c) they were not currently involved in any structured exercise programs out of school.

The pediatric obesity center submitted the study protocol to the school head teacher, which was approved by an ad hoc committee of parents and teaching staff at school. All participants and their parents provided written informed consent before participation. The study was conducted according to the World Medical Association Declaration of Helsinki and, as part of the HeLIOS research protocol, was approved by the local University and the local School Council Institute (03-2015/1227/B32c).

This quasi-experimental study consisted of a pre-test–post-test research design, with assessments at baseline (T_0_) and at the end (T_1_) of the program.

### 2.2. Treatment Program and PE Lessons

In addition to the official middle school PE curriculum, clinical participants followed a multicomponent treatment program focused on health-related motor activities, exercise training, and behavioral management skills. The exercise program required twice-weekly attendance over 7 months (from October 2018 to April 2019) and was held in the afternoon by a single teacher at the school gymnasium. All 2 h sessions included a wide variety of indoor and outdoor activities (i.e., sport games, circuits, and individual tasks), based on health-related fitness components (i.e., motor skills, flexibility, muscle strength, power, speed, agility, and aerobic fitness), aimed to increase participants’ activity levels through a range of enjoyable skill learning experiences and to improve their actual and perceived competence.

Children were encouraged to be physically active outside of the organized sessions and invited to fill out an activity diary weekly. The intervention program also included a weekly 30-min group meeting to teach children behavior management skills to improve their PA. Behavioral skill training sessions included review of PA diaries, goal-setting, problem-solving, self-monitoring, facilitative self-talk, self-rewarding, recruiting social support, and motivational activities. Drawing on Italian nutritional guidelines and recommendations [39,40], children were encouraged to engage in healthy eating behaviors (e.g., regular daily distribution of meals, reduced consumption of salt-rich foods and sugar-sweetened beverage, increased overall fruit and vegetable intake) at the time of admission to the hospital outpatient clinic, at the midpoint, and at the end of the intervention. The attendance of each child was recorded throughout the treatment program.

Nonclinical participants followed usual curricular PE lessons (2 h per week). The objectives of middle school PE were the development of physical fitness and health-related components (e.g., cardiorespiratory endurance, flexibility, muscular strength), skill-related components (e.g., coordination, agility, reaction time, balance, power, speed), sportspersonship, personal/social skills, and body awareness.

T_0_ and T_1_ assessments took place during four additional meetings a week before and a week after intervention, in September 2018 and in May 2019, respectively.

### 2.3. Measures

#### 2.3.1. Anthropometrics

Anthropometric measurements were recorded by an experienced pediatrician of the Center for Pediatric Obesity, according to the procedures of the International Society for the Advancement of Kinanthropometry [41]. Body weight to the nearest 0.1 kg and standing height to the nearest 0.1 cm were measured using a balance scale (Seca 761, Hamburg, Germany) and a calibrated stadiometer (Seca 220, Hamburg, Germany), respectively. BMI (kg/m^2^) was calculated from height and weight, and BMI centiles and z-scores were derived according to the CDC growth reference [38].

#### 2.3.2. Physical Activity

Participants completed the Physical Activity Questionnaire for Older Children (PAQ-C; [42]), which is a self-administered 7-day recall instrument developed for use with children aged 8–14 years. The PAQ-C assesses participation in different physical activities, as well as activity in PE class, recess, lunch break, after school, evenings, and at the weekend. It consists of 9 items scored on a five-point scale anchored by 1 (little or no activity) and 5 (very high levels of activity). Higher total scores indicate greater PA levels. Good internal consistency (Cronbach’s α = 0.79 to 0.89) and test–retest reliability (*r* = 0.75 to 0.82) have been established in children in grades 4 to 8 [42,43].

#### 2.3.3. Physical Fitness

Physical fitness was assessed using health-related tests (i.e., standing long jump, medicine ball throw, and 10 × 5 m shuttle-run), which have been reported to be reliable and valid measures in childhood and adolescence [44,45]:

- The standing long jump (SLJ) is aimed at evaluating lower-limb power. Children were permitted to perform a countermovement with the arms and legs before jumping horizontally. The test was performed twice, and the longest distance jumped was registered.
- The medicine ball throw (MBT) is intended to assess upper-limb power. Participants were asked to perform a two-hand overhead throw with a 2 kg medicine ball from a standing position with feet slightly apart. The highest value of two performance measures was recorded.
- The 10 × 5 m shuttle-run (SR) is meant to evaluate speed of movement, agility, and coordination. Children were instructed to run back and forth five times along a 10 m distance as quickly as possible. The test was performed twice, and the lowest sprint value was reported.

#### 2.3.4. Perceived Physical Ability

Children’s perceptions of strength, speed, and agility were evaluated using the Perceived Physical Ability Scale [46], which comprises 10 items pertaining to a positive scale (5 items) and a negative scale (5 items). Items are rated on a 5-point Likert scale ranging from 1 (very much) to 5 (not at all). Participants were asked to think about their performance when involved in physical activities and to indicate for each item the response that best represented their personal feelings. The positive scale assesses positive perceptions of physical ability (i.e., quick reaction and action, strength, and motor control), while the negative scale evaluates the perception of movement difficulty (i.e., lack of control, clumsiness, and muscle weakness). The items of the negative scale are reverse scored. Total scores on both scales can range from 0 to 25, with higher scores in each scale corresponding to higher perception of physical ability. Research has provided support for the scale as a valid and reliable measure in children [7,46] with Cronbach α = 0.81 and a spilt-half *r* = 0.82 [46].

### 2.4. Data Analysis

Data were preliminarily examined for missing values, distribution, and multivariate outliers [47]. No missing data or out-of-range values were observed. Inspection of skewness, kurtosis, histograms, and Q–Q plots indicated that data distributions did not substantially deviate from normality. Data are presented as mean ± SD. Pearson product–moment correlation coefficients (*r*) between variables were calculated for boys and girls at T_0_ and were interpreted according to Zhu’s [48] indications—that is, 0–0.19 = no correlation, 0.20–0.39 = low correlation, 0.40–0.59 = moderate correlation, 0.60–0.79 = moderately high correlation, and >0.80 = high correlation. Univariate analysis of variance (ANOVA) was performed to compare the anthropometric variable scores between the intervention group and the control group at baseline. A 2 (gender) × 2 (group: intervention vs. control) × 2 (time: T_0_ vs. T_1_) repeated-measures ANOVA design was applied to each dependent variable, and Bonferroni post hoc tests were employed to identify specific differences. Estimates of effect size (partial *η*^2^) are provided for significant findings, with values of 0.01, 0.06, and 0.14 indicating small, medium, and large effects, respectively [49]. In post hoc multiple comparisons, the effect sizes were calculated using Cohen’s *d* [50], whereby values of 0.20, 0.50, and 0.80 were considered small, moderate, and large, respectively. All analyses were conducted in SPSS version 26.0 (SPSS Inc., Chicago, IL, USA), and level of significance was set at *p* < 0.05.

## 3. Results

Baseline anthropometric characteristics of the children are shown in Table 1. Descriptive statistics for each variable by time of assessments, group, and gender are reported in Table 2.

Univariate ANOVA showed no differences between groups in the baseline variables, with the exception of height, *F*(1, 62) = 14.62, *p* < 0.001, *η*_p_^2^ = 0.19, and jump distance, *F*(1, 62) = 19.66, *p* < 0.001, *η*_p_^2^ = 0.24. Height and jumping performances of children in the experimental group were lower than those of their peers in the control group.

Repeated-measures ANOVAs yielded significant group × time interactions indicating that at T_1_ children in the intervention group exhibited significantly greater improvements in standing long jump, *F*(1, 60) = 6.86, *p* = 0.011, *η*_p_^2^ = 0.10, medicine ball throw, *F*(1, 60) = 5.51, *p* = 0.022, *η*_p_^2^ = 0.08, PA levels *F*(1, 60) = 14.58, *p* < 0.001, *η*_p_^2^ = 0.20, and perceived physical ability, *F*(1, 60) = 11.86, *p* = 0.001, *η*_p_^2^ = 0.17, compared with participants in the control group (see Table 2). Significant main effects by gender were found only for standing long jump, *F*(1, 60) = 7.79, *p* = 0.007, *η*_p_^2^ = 0.12, and medicine ball throw, *F*(1, 60) = 4.80, *p* = 0.032, *η*_p_^2^ = 0.07, with girls reporting lower values than boys.

Moreover, significant gender × group × time interactions were found. Post hoc analysis showed scores of boys and girls in the intervention group to be significantly larger in the standing long jump (boys, Cohen *d* = 1.214; girls, Cohen *d* = 0.373), medicine ball throw (boys, Cohen *d* = 0.108; girls, Cohen *d* = 0.743), perceived physical ability (boys, Cohen *d* = 0.373; girls, Cohen *d* = 0.196), and PA (boys, Cohen *d* = 0.313; girls, Cohen *d* = 0.823) than the scores of boys and girls in the control group. Girls in the control group (Cohen *d* = 0.190) and boys in the intervention group (Cohen *d* = 0.628) reduced speed time from baseline to post-test.

Correlation analysis indicated that BMI was positively related to the 10 × 5 m shuttle-run (boys, *r* = 0.42; girls, *r* = 0.40), and negatively associated with the standing long jump in boys (*r* = −0.41). Perception of physical ability correlated with PA levels (boys, *r* = 0.51; girls, *r* = 0.55), and standing long jump in girls (*r* = 0.39). Finally, PA was positively related to the standing long jump (*r* = 0.36) and medicine ball throw in boys (*r* = 0.38), and negatively associated with the 10 × 5 m shuttle-run in girls (*r* = −0.44). The magnitude of all these correlation coefficients ranged from low to moderate [48].

## 4. Discussion

This school-based intervention in overweight/obese 10–12-year-old boys and girls resulted in significant changes in almost all targeted variables. All participants completed a 7-month program, suggesting that the overall intervention was adequate and well received by the children. Participants in the clinical group showed improvements in PA levels, perceived physical ability, and performance in throwing and jumping tasks compared to their peers in the control group. Previous school-based studies have shown inconclusive results reporting small changes or no changes in PA (for reviews, see [32,51]). Our results, however, are consistent with those of previous uncontrolled pilot intervention studies conducted on overweight and obese children of similar age and nationality [5,36,52], which revealed significant changes in PA and health-related fitness tests. Our findings are also in line with the results of previous studies showing the effectiveness of school-based interventions in promoting PA and physical fitness among obese children (for a review, see [28]). From this standpoint, the increased PA levels in the intervention group could be linked to the improvement in actual motor competence, as found in previous studies [19,20]. Compared to the participants in the control group, children in the intervention group reported higher throwing and jumping actual performances at T1, whereas no change in sprint ability was noted. These findings concur with the conclusion of Watts et al.’s [53] review that exercise training may positively impact the muscular strength of obese children and adolescents. Contrary to our expectations and except for jumping and throwing performances, no gender differences were observed at T_0_ and T_1_, suggesting that overweight/obese boys and girls aged between 10 and 12 years do not differ in PA, sprint, and perceived motor abilities. The lower strength performance values observed in girls compared with boys were expected to emerge and could be explained by the notable gender differences in the development of strength around 11 to 13 years [54].

The correlations of BMI with shuttle run (positive) and standing long jump (negative) tests found in this study and in previous intervention studies [5,36] support the contention that overweight and obesity can have detrimental effects on motor performances [5,7,36,55,56]. Moreover, results of a pilot study with overweight and obese children showed positive associations between motor performance in the shuttle-run test and Harre circuit with unpleasant/dysfunctional states [36]. Therefore, as previous studies conducted in the context of school PE [57,58,59,60] suggest, an education environment that focuses on participants’ pleasant experiences is recommended in the promotion of PA.

One of the main findings in this study is the improved perception of physical ability of children in the intervention group. Given that perceived physical ability mediates the relationship between children’s motor competence and subsequent PA [19,21], these findings are not surprising. In fact, during the present program, children performed a wide variety of non-competitive activities and enjoyable exercises focused on mastering skills in a friendly supportive environment that enabled them to experience success and increase perceived competence, which are predictors of pleasant emotional states [61].

Although enjoyable activities based on skill improvement may not comply with classical training guidelines, research has shown that school-based interventions should be enjoyable and engaging rather than focusing solely on increasing PA [33]. Research also showed that enjoyment and participation in PA depends, to a large extent, on the individual’s perceived competence [62]. Interestingly, we found a moderate positive correlation between the perception of physical ability and PA in both boys and girls, suggesting that increasing confidence in personal performance can promote PA also in obese young people.

According to motivational theories emphasizing competence perception [18,35], it is important to increase actual and perceived physical competence of overweight and obese children to encourage them to adhere to PA programs [63]. Systematic reviews suggest that competence improvements occur even in the absence of weight loss [1] and that interventions focused on supervised exercise and PA can positively influence mental health of overweight children and adolescents [32]. However, research on psychological outcomes in school-based PA interventions for obese children is scarce [33,34], and it remains unclear whether enjoyment and competence perceptions during activity truly facilitate PA in the long term [62].

From this standpoint, a key strength of our study is the use of different measurements related to obesity, which is consistent with previous calls for the study of known factors that influence PA behaviors (e.g., successful motor experiences, improvement of physical fitness, increase of perceived competence) in interventions addressed to obese children [30]. Therefore, our findings could help develop effective school-based PA programs to improve the health of overweight/obese children. Another strength of our study was the adoption of a multicomponent program to explore the relationship between PA, physical fitness, and perception of physical ability in both clinical and nonclinical samples of overweight/obese children. This program represents an alternative approach with the adoption of enjoyable activities for skill improvement, which could be added to PE activities within the school environment. As Fairclough and Stratton [64] pointed out in their review, PE classes should be integrated with other school-based opportunities to contribute to young peoples’ daily PA and to counteract the rise in obesity.

Notwithstanding the promising findings, a limitation of our study is that it was a non-randomized controlled trial because the investigation was conducted in a real-world setting (i.e., the school context), with the cooperation of parents and professionals (i.e., pediatricians, dietitians, and teachers). In addition, we did not examine the potential influence of growth and weight loss on the participants’ results failing to collect long-term follow-up data (>12 months). Long-term follow-up studies of overweight/obese children could be useful to investigate the effects of different doses of PA on health outcomes and to further test the hypothesized relationship between PA and actual and perceived physical competences in overweight/obese children.

## 5. Conclusions

The results of this quasi-experimental study indicate that a multicomponent program focused on health-related motor activities, exercise training, and behavioral management skills is effective and can have important benefits for participants. The use of a multicomponent approach to weight management has proven successful in improving PA levels, physical fitness, and perceived physical ability among overweight/obese children. Our findings can have important implications for tailoring activity goals to individual participants and highlight the importance of a comprehensive healthy lifestyle program that includes physical, psychosocial, and behavioral factors. The initial phase of similar interventions should start with low levels of PA intensity that would then gradually increase. Weight-bearing motor tasks should be minimal to prevent participants’ withdrawal from PA. Clinical-family interaction is one of the key elements to include in the design or implementation of a school-based program aimed to prevent or reduce childhood obesity.

Overall, study findings may stimulate educators, trainers, and teachers to identify strategies and good practices targeting childhood obesity management by focusing on school initiatives based on a multicomponent approach and a variety of enjoyable physical experiences. This is expected to improve exercise adherence, physical fitness, and psychological well-being of overweight/obese children.

## Figures and Tables

**Table 1:** Baseline anthropometric characteristics of the participants.

	Control Group (*n* = 29)		Intervention Group (*n* = 35)	
Variable	Boys (*n* = 16)	Girls (*n* = 13)	Boys (*n* = 20)	Girls (*n* = 15)
	M ± SD	M ± SD	M ± SD	M ± SD
Age (years)	11.6 ± 0.2	11.5 ± 0.1	11.0 ± 0.6	11.0 ± 0.5
Height (cm)	154.1 ± 5.0	157.1 ± 8.2	147.7 ± 7.8	149.8 ± 7.0
Weight (kg)	61.3 ± 10.5	68.0 ± 13.5	58.6 ± 13.2	58.5 ± 10.4
BMI (kg/m^2^)	25.7 ± 3.5	27.4 ± 3.8	26.6 ± 4.0	25.9 ± 3.0
BMI *z*-score	1.8 ± 0.4	1.9 ± 0.4	2.0 ± 0.4	1.8 ± 0.3
BMI percentile	95.2 ± 3.7	96.1 ± 3.7	96.5 ± 3.1	96.1 ± 2.1

Note: BMI = body mass index.

**Table 2:** Descriptive statistics for the study variables before (T_0_) and after the intervention (T_1_).

	T_0_					
	Control Group			Intervention Group		
Variable	Boys	Girls	Total	Boys	Girls	Total
	M ± SD	M ± SD	M ± SD	M ± SD	M ± SD	M ± SD
SLJ (cm)	133.31 ± 24.99	108.46 ± 21.42	122.17 ± 26.26 *	97.95 ± 23.28	95.07 ± 13.99	96.71 ± 19.63 *
MBT (m)	4.98 ± 1.03	4.37 ± 0.81	4.70 ± 0.97 *	4.76 ± 0.98	4.40 ± 0.96	4.60 ± 0.97 *
SR (s)	23.14 ± 2.66	24.76 ± 2.00	23.87 ± 2.50	25.25 ± 2.64	24.73 ± 1.82	25.03 ± 2.31
PAL	2.08 ± 0.54	2.14 ± 0.58	2.11 ± 0.55 *	1.83 ± 0.44	2.01 ± 0.49	1.91 ± 0.46 *
PPA						
Positive scale	15.81 ± 3.75	16.38 ± 3.87	16.07 ± 3.74	16.25 ± 3.70	16.07 ± 2.40	16.17 ± 3.17
Negative scale	20.44 ± 2.73	20.08 ± 3.73	20.28 ± 3.16 *	18.55 ± 3.87	18.47 ± 3.02	18.51 ± 3.48 *
	**T_1_**					
SLJ (cm)	135.06 ± 23.71	110.77 ± 18.57	124.17 ± 24.50 *	108.50 ± 20.32	104.47 ± 15.31	106.77 ± 18.20 *
MBT (m)	5.12 ± 1.08	4.30 ± 0.86	4.75 ± 1.05 *	5.24 ± 1.14	5.01 ± 1.03	5.14 ± 1.08 *
SR (s)	22.82 ± 2.07	23.85 ± 1.65	23.28 ± 1.93	24.28 ± 2.51	24.19 ± 1.90	24.24 ± 2.24
PAL	2.13 ± 0.57	2.03 ± 0.51	2.09 ± 0.54 *	2.33 ± 0.69	2.53 ± 0.68	2.42 ± 0.68 *
PPA						
Positive scale	16.56 ± 3.92	17.08 ± 3.33	16.79 ± 3.61	17.80 ± 3.24	18.53 ± 2.70	18.11 ± 3.00
Negative scale	20.56 ± 3.41	21.69 ± 2.25	21.07 ± 2.95 *	21.75 ± 3.01	22.20 ± 2.86	21.94 ± 2.91 *

Note: SLJ = standing long jump; MBT = medicine ball throw; SR = 10 × 5 m shuttle-run; PAL = physical activity levels; PPA = perceived physical ability. * Significant group × time interactions *p* < 0.05.
